# Synthesis, characterization of two matrine derivatives and their cytotoxic effect on *Sf9* cell of *Spodoptera frugiperda*

**DOI:** 10.1038/s41598-020-75053-1

**Published:** 2020-10-22

**Authors:** Huiqing He, Xiangjing Qin, Fangyun Dong, Jingmin Ye, Chunbao Xu, Hanhui Zhang, Zhanmei Liu, Xiaojing Lv, Yuehua Wu, Xuhong Jiang, Xingan Cheng

**Affiliations:** 1 Institute of Natural Product Chemistry, College of Chemistry and Chemical Engineering, Zhongkai University of Agriculture and Engineering, Guangzhou, 510225 Guangdong China; 2 CAS Key Laboratory of Tropical Marine Bio-Resources and Ecology, Guangdong Key Laboratory of Marine Materia Medica, South China Sea Institute of Oceanology, Chinese Academy of Sciences (CAS), Guangzhou, 510301 China; 3 Department of Chemical and Biochemical Engineering, Western University, London, Ontario N6A5B9 Canada; 4 Institute of Plant Health, Zhongkai University of Agriculture and Engineering, Guangzhou, 510225 Guangdong China

**Keywords:** Biochemistry, Biotechnology, Chemical biology, Structural biology, Environmental sciences

## Abstract

The invasion of *Spodoptera frugiperda* has imposed a serious impact on global food security. Matrine is a botanical pesticide with a broad spectrum of insecticidal activity which was recommended for controlling *Spodoptera frugiperda*. In order to discover effective insecticide for *Spodoptera frugiperda*, two matrine derivatives modified with carbon disulfide and nitrogen-containing groups were systhesized. And their inhibition activities on *Sf9* cell were evaluated. The structural configuration of compounds were characterized by IR, HPLC, MS, NMR and XRD, with yields of 52% and 65%, respectively. The IC_50_ of the two newly synthesized compounds on *Sf9* cell reduced to 0.648 mmol/L and 1.13 mmol/L, respectively, compared with that of matrine (5.330 mmol/L). In addition, microscopic observation of *Sf9* cell treated with the compounds showed that the number of adherent cells decreased, the cells shrunk, vacuolated and apoptotic bodies appeared. The two newly synthesized compounds exhibited better inhibitory effect on *Sf9* cell than that of the parent matrine, suggesting that the positive effect of the introduction of 1-pyrrolidinecarbodithioate and diethylcarbamodithioate groups to matrine. The morphological observation of *Sf9* cell induced by derivatives indicated that apoptosis induction may be a mechanism that inhibits insect cell proliferation and exerts insecticidal effect.

## Introduction

The invasion of *Spodoptera frugiperda* (*S. frugiperda*) with strong resistance and migration ability has imposed a serious impact on global food security. Relevant reports and studies have shown that it is an omnivorous pest with great appetite which may result in reduced or lost harvest^1^. With the help of air currents and monsoons, *S. frugiperda* which were divided into corn-strain and rice-strain, can spread across continents^2–4^. Therefore, it is extremely difficult to make the prevention and control of *S. frugiperda* due to its severe harm, fast mobility, wide spreading range, strong drug resistance, etc.

The severity of the insect pests has aroused great attention from the people, and many effective methods for controlling *S. frugiperda* were used^5^. Due to the resistance of *S. frugiperda*, many traditional synthetic insecticides have not been recommended for use, including organophosphorus, pyrethroids, carbamates and so on, among which its resistance to pyrethroid pesticide has reached hundreds of times^6,7^. Researchers have begun exploring natural ingredients as active compounds for the prevention and control of *S. frugiperda*. Studies on the insecticidal activities of natural compounds and their derivatives against *S. frugiperda* have been reported, such as botanical essential oils and sesquiterpenoids. It was found that methylchavirol has an effect on development and metabolism of the insect, while pepper oil has an effect on embryo development^8,9^. And the examination of 18 sesquiterpenoids showed that the drimanic family with C-9 carbonyl and C-8 and C-9 epoxides have significant antifeedant activity against *S. frugiperda* and *Epilachna paenulata*, suggesting the important of structure–activity relationship for exploring pesticides^10^. However, a few active ingredients have been investigated to effectively control *S. frugiperda*, so there is an urgent need to develop highly effective biological pesticides.

It has been reported that new amides and Bt toxins have a potential to control *S. frugiperda*^11–13^. As a biological pesticide with lactam structure, matrine has been recommended as a biological pesticide for controling *S. frugiperda*. However, the promotion of matrine as a biological pesticide is severely hampered by insignificant activity, slow efficacy and low bioavailability. It is a common method to develop new pesticides by modifying the structure of natural compounds^14^. A series of novel quinolinomatrine derivatives which were designed and synthesized from natural product insecticide exhibited good insecticidal and acaricidal activities^15^. The introduction of trifluoroethoxy-containing carbonyl thiourea, trifluoroethyl ether, propargyl ether and diacylhydrazine bridge groups can significantly improve the insecticidal activity of anthranilamide^16–19^. According to the special insecticidal mechanism of pyridalyl against lepidopterous larvae, 1,1-dichloropropene derivatives bearing structurally diverse substituted heterocycle rings that replace the pyridine ring of pyridalyl have been designed and synthesized, and the compounds displayed significant insecticidal activity against p*rodenia litura* and *diamondback moths*^20^*. *In addition, our previous studies showed that introducing carbon disulfide and cyclohexylamine groups can improve the toxicity of matrine to *Lipaphis erysimi* and *Mulberry Root-Knot Nematode*, while the introduction of indole and cyclohexylamine groups can increase the inhibition activity of matrine on *Sf9* cell^21,22^.

Studying the cytotoxicity of insecticides is helpful to further study insecticidal mechanism. For example, β-Asarone extracted from Acorus calamus Linn exhibited inhibitory activity on *Sf9* cell through apoptosis induction^23^. Vip3Aa protein showed a high toxicity against lepidopteran insect larvae and promoted apoptosis of *Sf9* cell through mitochondrial dysfunction^24,25^. And synthesized proVip3Aa toxin caused cell disruption and death via apoptosis^26^. Moreover, recombinant virus AcMNPV-Ac34-EGFP could activate the JNK apoptotic signaling pathway to inhibit the proliferation of *Sf9* cell, and curcumin could induce nucleophagy by blocking the activation of PI3K/AKT/TOR pathways^27,28^. Research on the insecticidal mechanism is of great significance to the development of insecticides.

Carbon disulfide is used as an intermediate for pesticides, and nitrogen-containing substances are used as active modification groups^29,30^. Particularly, nitrogen-containing heterocycles proved to have strong systemic conductivity and unique biological activity^31,32^. This research aims to improve the inhibitory activity of matrine on *S. frugiperda* cells by modifing the structure of matrine. Two new compounds were designed and synthesized by introducing 1-pyrrolidinecarbodithioate and diethylcarbamodithioate groups, and their insecticidal effects on *Sf9* cell were also explored in vitro.

## Results

### Chemical synthesis

**Figure 1:**
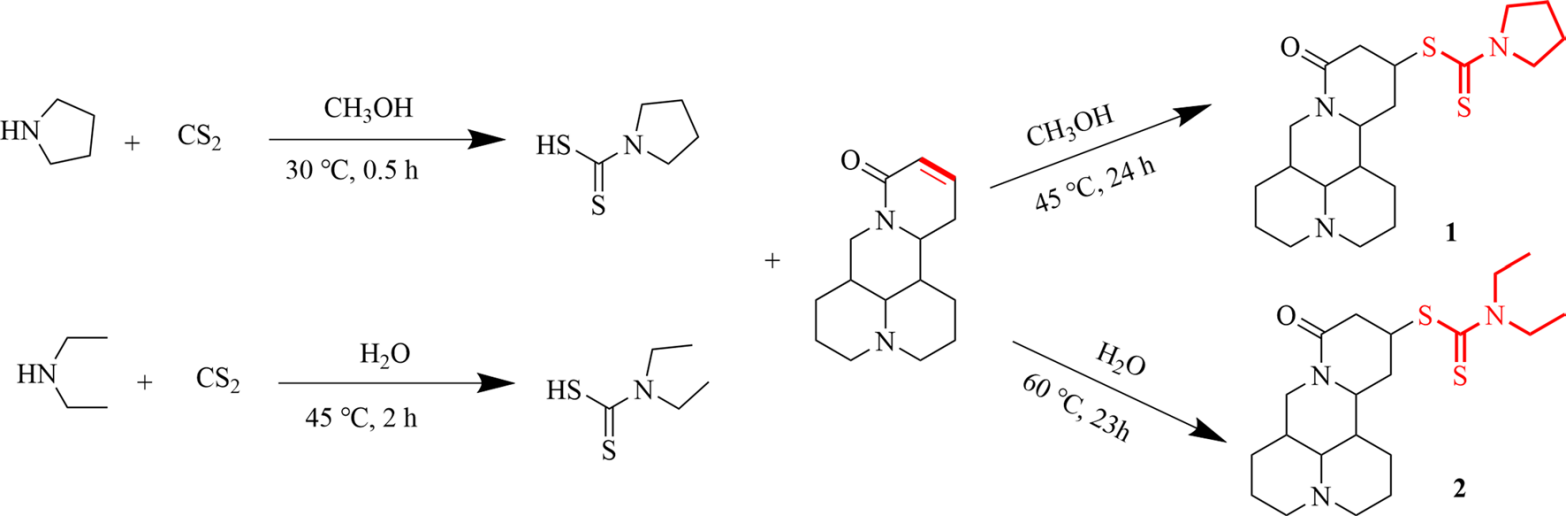
Synthesis of matrine derivative compounds 1 and 2.

The synthesis of two compounds were carried out by two nucleophilic additions in one-pot (Fig. 1). Firstly, the amine reacted with carbon disulfide to synthesize intermediate compounds (1-pyrrolidinecarbodithioic acid, diethylcarbamodithioic acid). Secondly, the intermediate compounds reacted with sophocarpine to produce the target products. Experiments showed that carbon disulfide could also react with sophocarpine at the first step, but the yields were low. Compounds 1 and 2 were reacted in ethanol and ultrapure water, respectively. The unreacted reactant and solvent were separated, and crystals were obtained after purification with the yields of 52% and 65%, respectively.

### Spectroscopic characterization

**Figure 2:**
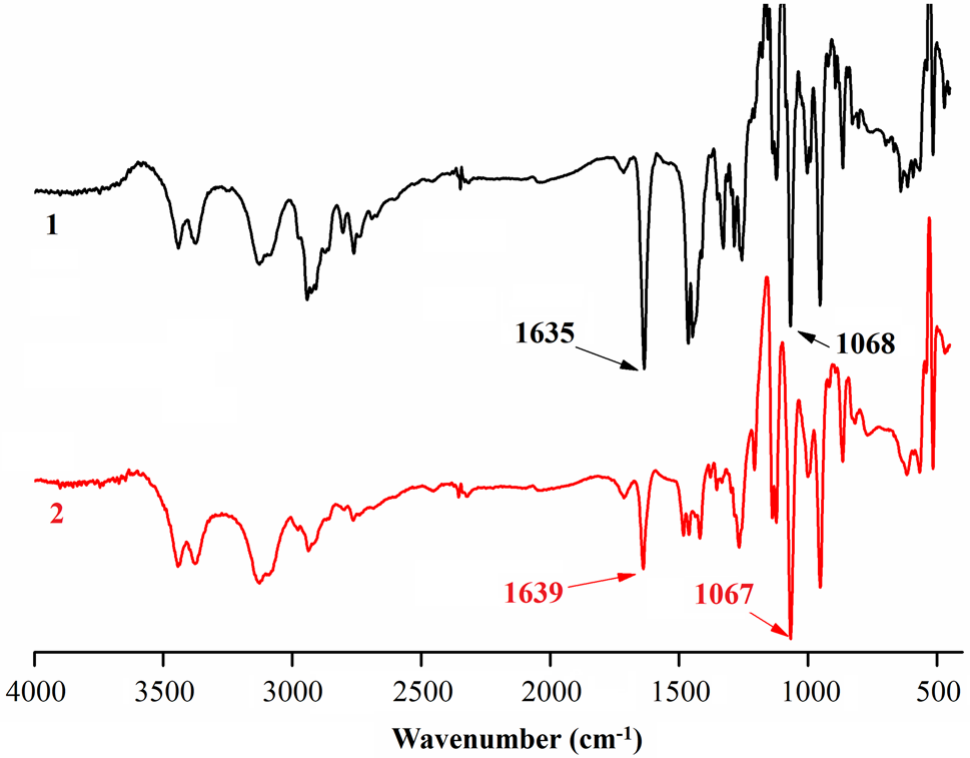
IR spectra of the obtained matrine derivative compounds 1 and 2.

The IR spectra of compounds 1 and 2 are shown in Fig. 2. The strong peak at 1635 cm^−1^ and 1639 cm^−1^ were assigned to carbonyl group absorption. Additionally, due to the introduction of the groups, the peak at about 1600 cm^−1^ which was assigned to carbon–carbon double bond of sophocarpine disappeared. And the characteristic bands at about 1068 cm^−1^ and 1067 cm^−1^ can be assigned to the ν(C=S) absorption. It indicates that the active groups were introduced into matrine.

**Figure 3:**
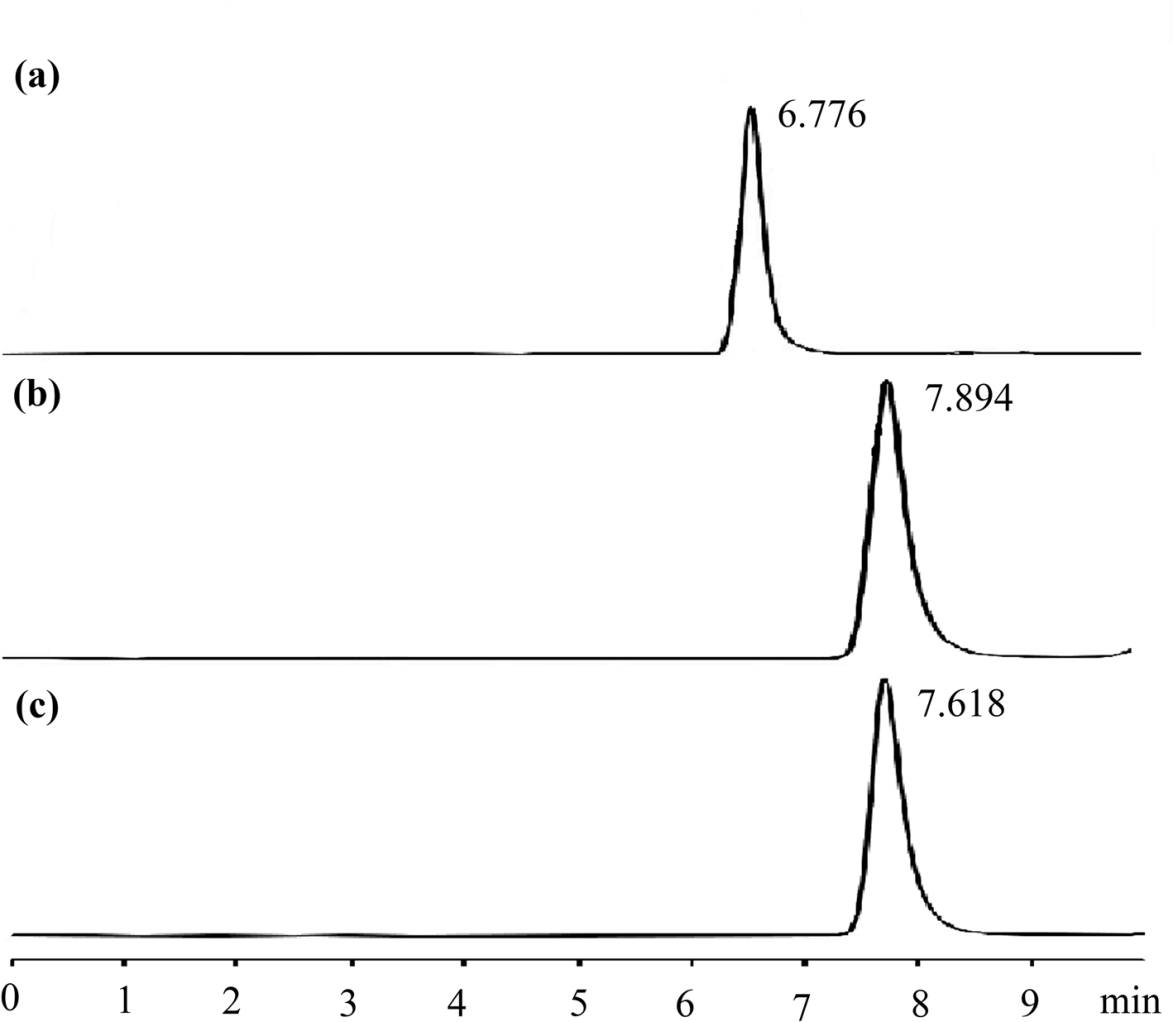
HPLC chromatograms of sophocarpine (**a**), and compounds 1 (**b**) and 2 (**c**).

HPLC chromatograms of sophocarpine, compounds 1 and 2 are shown in Fig. 3a–c. The retention time of sophocarpine, compounds 1 and 2 were at 6.776, 7.894 and 7.618 min, respectively. Combined with IR spectra analysis, active groups were introduced into matrine, and the obtained compounds were pure.

The mass spectrum of compounds 1 ~ 2 are showed in Supplementary Figs. 1S and 2S online. The molecular formula were determined as C_20_H_31_N_3_OS_2_ and C_20_H_33_N_3_OS_2_ on the basis of ESI–MS ion peaks at m/z 393.19 ([M + H]^+^, calcd 394.8) and 395.21 ([M + H]^+^, calcd 396.7) respectively.

The composition of compounds 1 and 2 are determined by NMR spectroscopy, as shown in Supplementary Figs. 3S–6S online. The C13 of compounds 1 and 2 showed two triplet at about 2.8 ppm in ^1^H NMR spectroscopy, and a singlet at about 42 ppm in the ^13^C NMR spectroscopy. The chemical shift peak at about 166 ppm was attributed to the carbon atom of carbonyl from matrine. Even if the chemical structure difference is very small, the peak can be separated in carbon and hydrogen spectrum. The chemical shift of carbon–sulfur double bond (C=S) in compounds 1 and 2 were observed at 190.91 ppm and 193.53 ppm, respectively. And the multiplets at 1.94–2.01 ppm in the ^1^H NMR spectroscopy and singlet at 20.89 ppm in the ^13^C NMR spectroscopy can be assigned to the C24, 25 of the compound 1. The C24, 25 of compound 2 showed a triplet at 1.27 ppm in ^1^H NMR spectrum, and a singlet at 11.71 ppm and 12.66 in the ^13^C NMR spectroscopy, respectively. This is because the pyrrolidinyl group is cyclic, while the diethylamine group is chain. Thus, the FT-IR and LR–ESI–MS analysis results both demonstrate the successful synthesis of matrine derivatives.

### Structural descriptions

X-Ray diffraction patterns of compounds 1 and 2 were collected by a XtaLAB PRO MM007HF diffractometer. The crystals were kept at 100.00 (10) K during data collection. Table 1 summarized the pertinent crystallographic data and refinement details of compounds 1 ~ 2. The crystal data of these two substances have never been published previously.

**Table 1:** Crystallographic data and details of refinement for compounds 1 ~ 2.

Matrine derivatives	Compound 1	Compound 2
Empirical formula	C_20_H_31_N_3_OS_2_	C_20_H_37_N_3_O_3_S_2_
Formula weight	393.62	431.67
Crystal system	Monoclinic	Monoclinic
Space group	P2_1_	P2_1_
a/Å	12.1713 (2)	14.5071 (4)
b/Å	5.27090 (10)	5.21060 (10)
c/Å	15.5687 (3)	14.7222 (4)
α/°	90	90
β/°	101.992 (2)	95.066 (2)
γ/°	90	90
Volume/Å^3^	976.99 (3)	1108.51 (5)
Z	2	2
μ/mm^−1^	2.576	2.381
F(000)	426.3	470.6
Reflections collected	9331	9965
Independent reflections	3685 [Rint = 0.0228]	4340 [Rint = 0.0344]
Goodness-of-fit on F^2^	1.030	1.028
Final R indexes [I ≥ 2σ (I)]	R_1_ = 0.0440, wR_2_ = 0.1290	R_1_ = 0.0428, wR_2_ = 0.1234
Final R indexes [all data]	R_1_ = 0.0442, wR_2_ = 0.1292	R_1_ = 0.0455, wR_2_ = 0.1241

The crystallographic data of compounds 1 and 2 have been deposited with the Cambridge Crystallographic Data Centre as supplementary publication number CCDC 1576162 and 1576161, respectively. Some key crystallographic data of compounds 1 and 2 were provided in Tables 1 and 2. In addition, our previous work had reported the crystallographic data of sophocarpine^28^. Therefore, the single crystal diffraction analysis will be compared with the previous data. After the Michael addition reaction, the carbon–carbon double bonds of sophocarpine disappeared, causing the bond length in the lactam ring to stretch, and the bond angle also changed to varying degrees which also indicated that the pyrrolidine and diethylamine groups were successfully introduced.

**Table 2:** Bond lengths (Å) and angles (°) of sophocarpine and compounds 1 ~ 2.

Sophocarpine			
C12–C13	1.4925 (16)	C12–C13–C14	120.73 (10)
C13–C14	1.3229 (18)	C13–C14–C15	121.78 (11)
C14–C15	1.478 (16)	N16–C15–C14	117.84 (10)

Compound 1			
C12–C13	1.526 (3)	C12–C13–C14	108.96 (19)
C13–C14	1.532 (3)	C13–C14–C15	115.27 (19)
C14–C15	1.518 (3)	N16–C15–C14	118.4 (2)
S19–C13	1.812 (2)	C12–C13–S19	110.41 (17)
S19–C20	1.782 (2)	C13–S19–C20	103.98 (11)
S21–C20	1.669 (2)	N22–C20–S21	123.79 (19)
N22–C23	1.482 (3)	C23–N22–C26	111.51 (18)
C20–N22	1.332 (3)	C25–C26–N22	103.2 (2)
C25–C24	1.526 (4)	C24–C23–N22	102.53 (19)
C23–C24	1.515 (4)	C20–N22–C23	123.1 (2)

Compound 2			
C12–C13	1.524(4)	C12–C13–C14	109.2 (3)
C13–C14	1.527 (4)	C13–C14–C15	114.2 (3)
C14–C15	1.517 (4)	N16–C15–C14	118.3 (3)
S19–C13	1.817 (3)	C12–C13–S19	113.1 (2)
S19–C20	1.787 (3)	C13–S19–C20	103.86 (15)
S21–C20	1.667 (3)	N22–C20–S21	124.5 (3)
N22–C23	1.471 (4)	C23–N22–C26	115.1 (3)
C20–N22	1.339 (4)	C25–C26–N22	112.6 (3)
C23–C24	1.517 (5)	C24–C23–N22	112.5 (3)
O18–H3	1.847	C20–N22–C23	121.1 (3)
O2W–H1	2.032	O1W–H1	0.842
O2W–H2	2.157	O1W–H2	0.740
O2W–H3	0.867	O1W–H4	2.009
O2W–H4	0.788		

**Figure 4:**
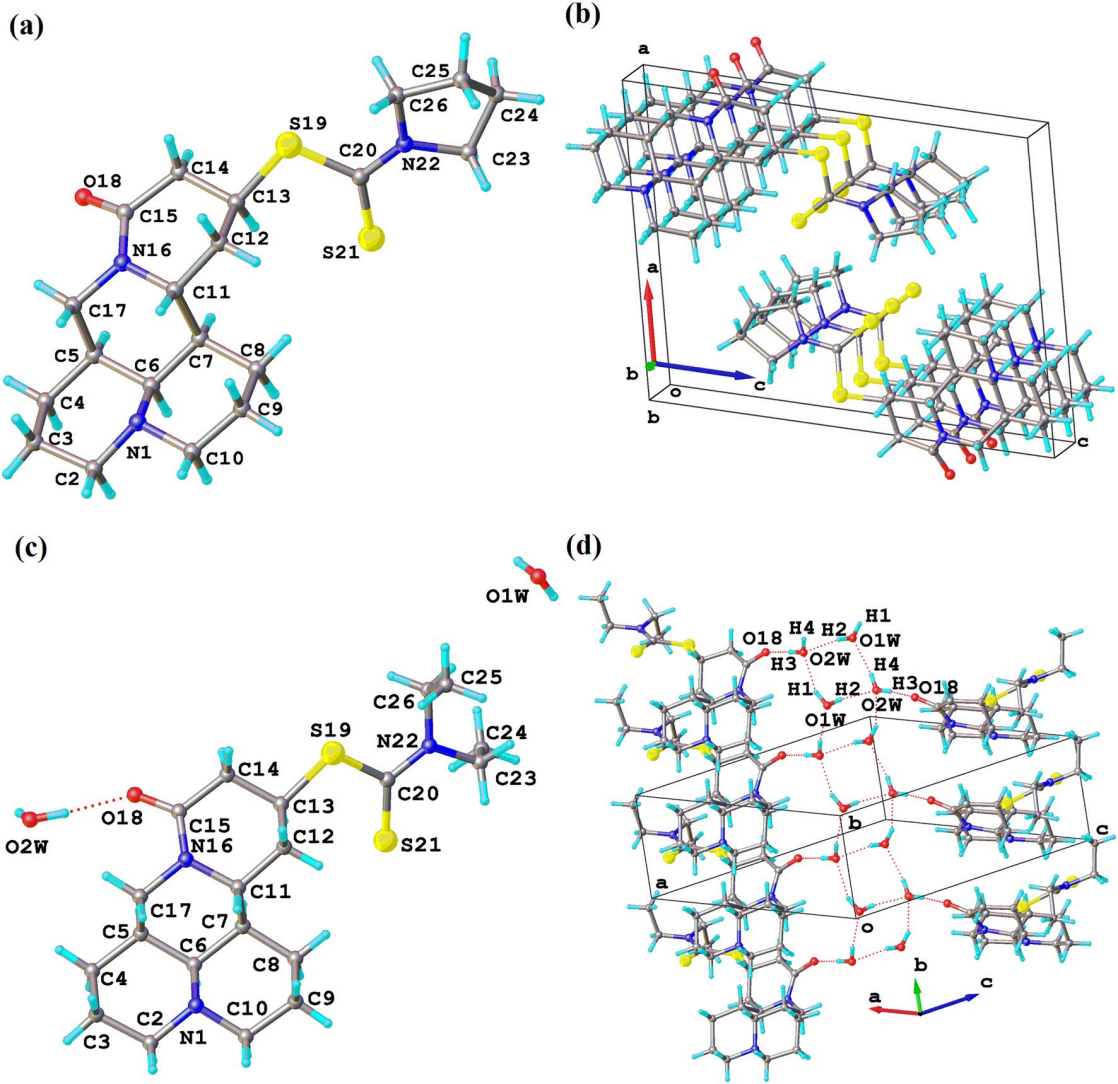
Crystal structure (**a**, **c**) and the pack pictures (**b**, **d**) of compounds 1 and 2.

In compound 1, matrine and carbon disulfide were linked by S19-C13 with the bond length of 1.812 (2) Å, forming the bond angle of C12-C13-S19 110.41 (17)°. In addition, carbon disulfide and pyrrolidine were linked by C20-N22 with the bond length of 1.332 (3) Å, forming the bond angle of C20-N22-C23 [123.1(2)°] (Fig. 4a). Similarly, the bond lengths of S19-C13 and C20-N22 of compound 2 were 1.817 (3) Å and 1.339 (4) Å, forming the bond angles of C12-C13-S19 [113.1 (2)°] and C20-N22-C23 [121.1(3)°] (Fig. 4c). In the pyrrolidine group of compound 1, the bond angles of C23-N22-C26 and C25-C26-N22 were 111.51 (18)° and 103.2 (2)°, respectively. However, in the compound 2 with a chain diethylamine, the bond angles of C23-N22-C26 and C25-C26-N22 were 115.5 (3)° and 112.6 (3)°, respectively.

The molecules were arranged in order to form a crystal structure, and there was no hydrogen bond between the molecules in compound 1 (Fig. 4b). The oxygen atom in the molecule of compound 2 and the hydrogen atom in the molecule of H_2_O formed an O18–H3 bond with the bond length of 1.847 Å. And it was found that every two molecules were linked by three H_2_O molecules, and every four H_2_O molecules formed an eight-membered ring through hydrogen bonding. The eight-membered ring were alternately connected by H_2_O containing O1W and O2W atoms, forming the bond with O1W–H1 [0.842 Å], O1W–H2 [0.740 Å], O1W–H4 [2.009 Å] and O2W–H1 [2.032 Å], O2W–H2 [2.157 Å], O2W–H3 [0.867 Å], O2W-H4 [0.788 Å], respectively (Table 2). Finally, compound 2 was connected to the H_2_O molecule and folded into an ordered supramolecular network structure (Fig. 4d).

### Cytotoxic effect on *Sf9* insect cell

**Figure 5:**
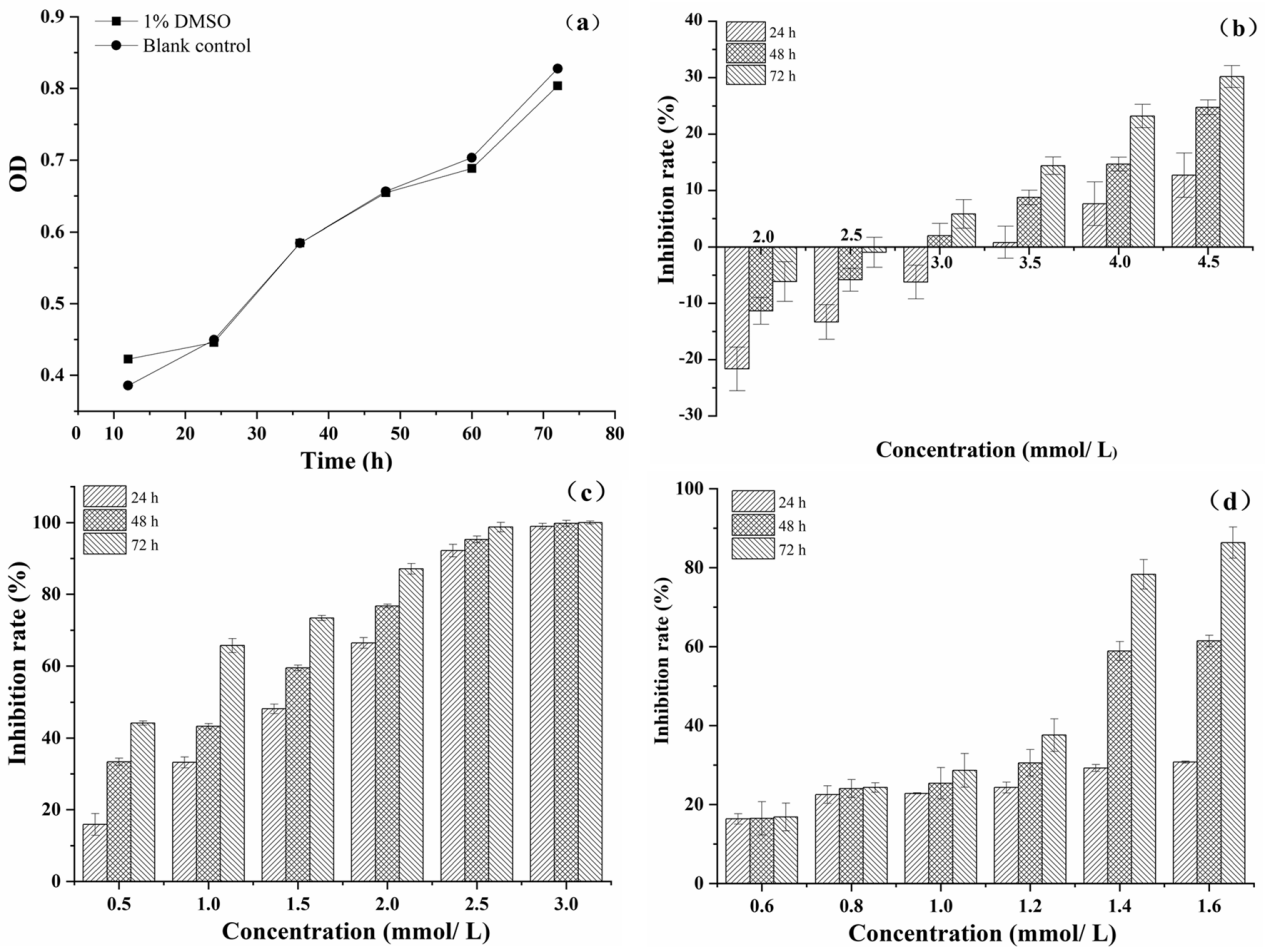
The growth curve of *Sf9* cell treated with 1.0% DMSO compared with the blank control group (**a**), inhibition rate of *Sf9* cell after treatment with matrine (**b**), and compounds 1 (**c**) and 2 (**d**).

**Figure 6:**
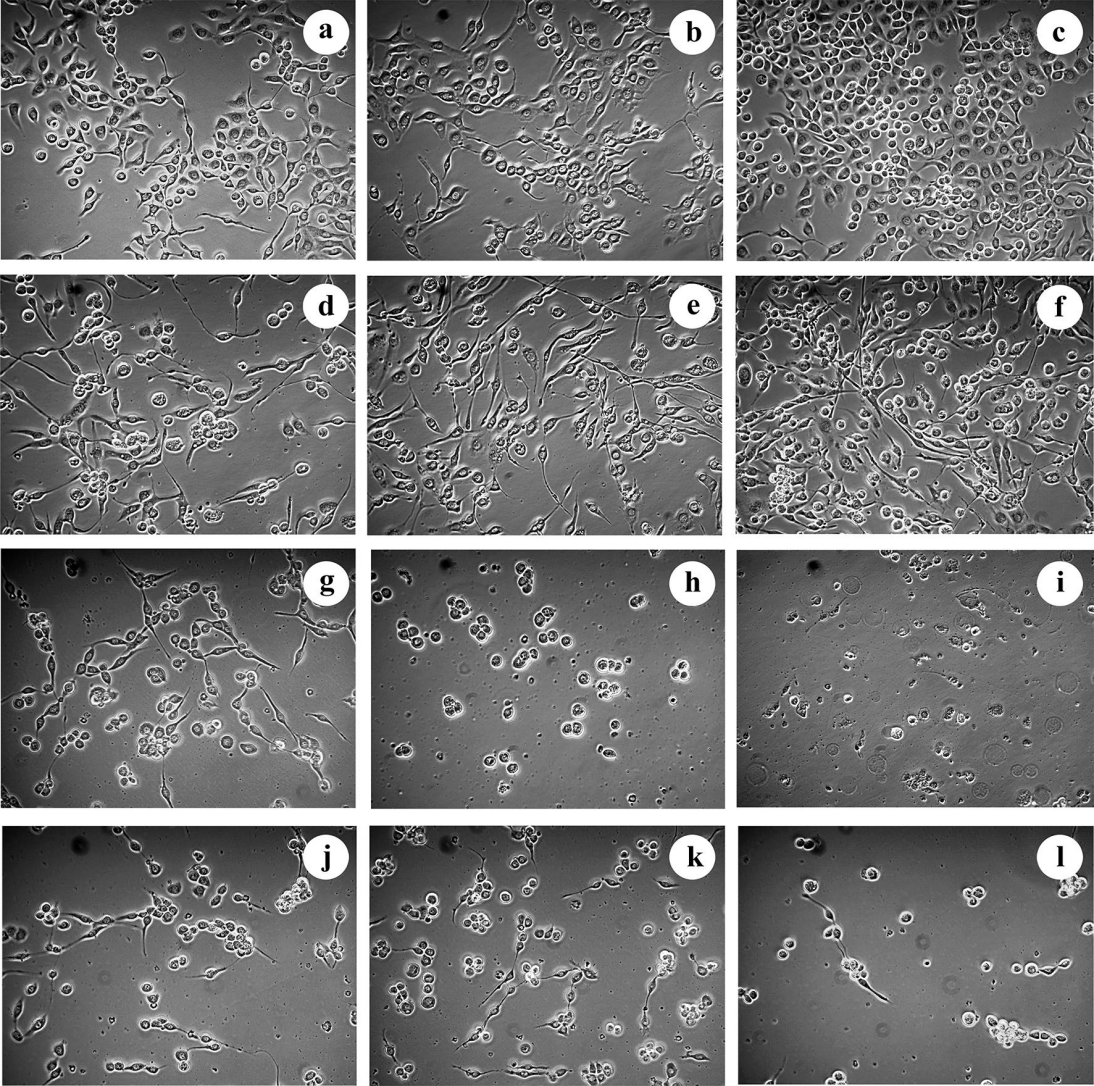
Analysis of proliferation and cell morphological changes (200 ×) of *Sf9* cell in 1.0% DMSO as the control (**a**–**c**) or induced by matrine (**d**–**f**), compound 1 (**g**–**i**), or compound 2 (**j**–**l**) at 24, 48 and 72 h.

Figure 5 displayed the growth curve of *Sf9* cell treated with 1.0% DMSO and blank control group (Fig. 5a), from which it can be seen that the growth curve of *Sf9* cell treated with 1.0% DMSO was almost replicated that of the blank control group. It was observed that the number of cells entered a logarithmic growth phase in 24 h. The observation and analysis of Inverted Phase Contrast Microscopy (IPCM) showed that the *Sf9* cell treated with 1.0% DMSO were in a good condition at 24, 48, and 72 h, showing complete adherence, vigorous cell proliferation, and in a long or fusiform shape (Fig. 6a–c). Thus, it indicates that 1.0% DMSO can be used as a solvent for the test compounds.

The effects of matrine on *Sf9* cell are showed in Fig. 5b. Matrine promoted the proliferation of *Sf9* cell at a concentration less than 3.0 mmol/L, but inhibited the proliferation at a concentration above 3.0 mmol/L. The inhibition rate of matrine at 4.5 mmol/L was 30.20% at 72 h. Moreover, the inhibition rate gradually improved with increasing treatment time and concentration, suggesting that inhibition activity of matrine against *Sf9* cell were concentration-dependent and time-dependent. The half maximal inhibitory concentration (IC_50_) of matrine to *Sf9* cell was 8.586, 5.402 and 5.336 mmol/L at 24 h, 48 h and 72 h, respectively (Table 3). Compared with the blank control group, most of the cells treated with matrine were in a normal growth state at 24 h and 48 h, while the number of adherent cells was reduced at 72 h, when the cell membrane was translucent and some cells shrank in size (Fig. 6d–f).

**Table 3:** IC_50_ of matrine and compounds 1 and 2 against *Sf9* cell.

Compounds	IC_50_ (mmol/L)		
	24 h	48 h	72 h
Matrine	8.586	5.402	5.336
Compound 1	1.271	0.938	0.648
Compound 2	4.646	1.389	1.130

As can be clearly seen from Fig. 5c, compound 1 showed remarkable inhibitory effects against *Sf9* cell. The 24 h inhibitory rate of the compound 1 on *Sf9* cell was above 90% at 2.5 mmoL/L, and the 24 h inhibition rate reached about 100% when the concentration was 3.0 mmoL/L. Compared with matrine, the IC_50_ of compound 1 was greatly reduced, being 1.271, 0.938 and 0.648 mmol/L at 24 h, 48 h and 72 h, respectively (Table 3). The results of IPCM observation of the cells treated with 2.5 mmoL/L compound 1 for 24 h showed that the number of adherent cells decreased sharply, and the volume of the cells shrank to a circular shape, although a small number of cells were still dividing in Fig. 6g. The cells shrank and shed at 48 h, when the cell membrane became brighter, the cytoplasm color darkened, and apoptotic bodies appeared, indicating that apoptosis occurred after 48 h of induction (Fig. 6h). Furthermore, the cells were severely vacuolated, and there were no normal cells in the visual field at 72 h (Fig. 6i).

Compound 2 at medium–low concentration (less than 1.4 mmol/L) showed a low inhibitory effect on *Sf9* cell, and the 24 h inhibition rate was less than 50%. However, the inhibition rate reached 86.34% when the cells were treated with compound 2 at 1.6 mmol/L for 72 h (Fig. 5d). And the IC_50_ of compound 2 against *Sf9* cell attained 4.646, 1.389 and 1.130 mmol/L at 24 h, 48 h and 72 h, respectively (Table 3). Combined with IPCM analysis, compound 2 significantly inhibited the proliferation of *Sf9* cell. The number of adherent cells decreased, and the volume of some cells shrank and became round after treated with compound 2 at 1.6 mmol/L for 24 h (Fig. 6j). The number of adherent cells continued to decline gradually, and most of the cells became round and shrank after induction for 48 and 72 h (Fig. 6k–l). In addition, the cells exhibited vacuolated and chromatin-colored, indicating occurance of apoptosis at 72 h (Fig. 6l).

In summary, compared with matrine, both compounds 1 and 2 showed significant inhibitory effects on *Sf9* cell, suggesting that the introduction of 1-pyrrolidinecarbodithioate and diethylcarbamodithioate groups can effectively enhance the inhibitory effect of matrine on *Sf9* cell. Although the structures of the compounds 1 and 2 are similar, differing only in the nitrogen function groups introduced, i.e., heterocyclic nitrogen ring in compound 1 and a chain structure of amine in compound 2, the activity of the compound 1 was found to be much higher than that of compound 2, likely owing to the unique biological activity of the nitrogen-containing heterocycles^31,32^.

## Conclusions

Two matrine derivatives containing carbon disulfide and nitrogen-containing groups were successfully synthesized, and their structures were confirmed by various structure characterization techniques. The in vitro activity tests indicated that two matrine derivative compounds 1 and 2 exhibited significant cytotoxic activity against *Sf9* cell, suggesting the introduction of 1-pyrrolidinecarbodithioate and diethylcarbamodithioate groups to matrine molecular structure could greatly increase the insecticidal activity of matrine significantly. The results of this work provides a new direction for controlling *S. frugiperda* and a method for improving pesticide activity of matrine through structural modification.

## Materials and methods

### Materials

Sophocarpine (purity ≧ 98%) and matrine (purity ≧ 98%) were purchased from Baoji Fangsheng Biological Development Co., Ltd. Pyrrolidine was purchased from Shanghai Macklin Biochemical Co., Ltd. Carbon disulfide, petroleum ether, and anhydrous methanol were purchased from Tianjin Damao Chemical Reagent Factory. Diethylamine was purchased from Shanghai Aladdin Biochemical Technology Co., Ltd. Absolute ethanol, ethyl acetate and dichloromethane were purchased from Tianjin Kaitong Chemical Reagent Co., Ltd., Tianjin Beilian Fine Chemical Development Co., Ltd., and Tianjin Best Chemical Co., Ltd., respectively. Fetal bovine serum (FBS), antibiotics and phosphate buffer solution (PBS) were purchased from Gibco. Dimethyl sulfoxide (DMSO) and Thiazole blue (MTT) were purchased from SIGMA and Guangzhou Qiyun Biotechnology Co., Ltd., respectively.

### Analytical methods

Infrared spectra were measured by Spectrum 100 (PerkinElmer) with KBr disk. Mass spectral data were collected by Bruker amaZon speed. ^1^H NMR and ^13^C NMR spectra were obtained on a Bruker AVANCE III HD 500 MHz spectrometer (Bruker). X-Ray diffraction patterns of compounds were collected using an XtaLAB PRO MM007HF diffractometer. An Agilent HPLC 1200 was used for HPLC analysis under the main chromatographic conditions, including a CNW Athena C18-WP (4.6 mm × 250 mm, 5 µm) column, a detection wavelength of 220 nm, and a mobile phase of 10:80:10 (v/v/v) ethanol/Acetonitrile/KH_2_PO_4_ (ϕ = 0.3%) at a flow rate of 1.0 mL min^−1^.

### Synthesis of compound 1

Anhydrous methanol (2.00 mL) and carbon disulfide (1.00 mL) were added into a 50 mL three-neck flask equipped with a condensation and reflux device, and stirred vigorously at 30 °C. Pyrrolidine (0.42 mL) was slowly added into the mixture. The reaction was violent, and white smoke was observed. White smoke disappeared after 0.5 h. Sophocarpine (0.31 g) and anhydrous methanol (2.00 mL) were added with stirring and continued reacting for 24 h at 45 °C. Finally, a slightly black and yellow viscous product was obtained. The reaction mixture was collected after adding 10 mL of anhydrous methanol and filtered by Buchner funnel. The concentrated filtrate was separated by column chromatography and eluted with ethanol: ethyl acetate (1:10, v/v). After the initial product was obtained, it was purified by crystallization with mixture of ethanol-petroleum ether (1:4, v/v).

### Synthesis of compound 2

Purified water (2.00 mL) and of diethylamine (1.00 mL) were added into a 50 mL three-necked flask equipped with a condensing and refluxing device, and stirred vigorously at 45 °C. Carbon disulfide (0.60 mL) was slowly added into the mixture. A large amount of white smoke and a large amount of white solids were observed in the flask after 2 h. Sophocarpine (0.615 g) and purified water (2.00 mL) were added into the three-neck flask to react at 60 °C for 23 h. A yellow viscous material was obtained. The reaction mixture was obtained after adding 10 mL of dichloromethane and filtered on a Buchner funnel. The concentrated filtrate was separated by column chromatography and eluted with ethyl acetate: methanol (10:1, v/v). The target product obtained by column chromatography was evaporated and concentrated, and crystallized in petroleum ether.

### Data for compound 1

Yield: 52%, Colorless. Selected IR data (KBr disk, cm^−1^): ν (C=O, Carbonyl) 1635 s; ν (C=S, Carbon–sulfur double bond) 1068 s. ^1^H NMR (500 MHz, CDCl_3_) δ 1.22–1.53 (m, 4 H), 1.53–1.76 (m, 4 H), 1.94–2.01 (m, 4 H), 2.05 (t, J = 6.6 Hz, 2 H), 2.14–2.27 (m, 4 H), 2.63 (tt, J = 6.7, 17.4 Hz, 4 H), 2.78 (dd, J = 5.2, 17.3 Hz, 1 H), 3.10 (t, J = 12.7 Hz, 1 H), 3.59 (t, J = 6.9 Hz, 2 H), 3.86–4.02 (m, 4 H), 4.37 (dt, J = 4.4, 12.7 Hz, 1 H). ^13^C NMR (126 MHz, CDCl_3_) δ 20.89, 21.31, 24.43, 26.18, 27.84, 31.97, 35.75, 37.80, 41.97, 42.57, 50.94, 52.12, 55.04, 57.47, 64.17, 166.87, 190.91. LR–ESI–MS: calculated for C_20_H_31_N_3_OS_2_ [M + H]^+^: 393.2, found: 394.8. HPLC: 7.894 min.

### Data for compound 2

Yield: 65%, Colorless. Selected IR data (KBr disk, cm^-1^): ν (C=O, Carbonyl) 1639 s; ν (C=S, Carbon–sulfur double bond) 1067 s. ^1^H NMR (500 MHz, CDCl_3_) δ 1.27 (t, J = 6.8 Hz, 6 H), 1.39–1.55 (m, 4 H), 1.55–1.78 (m, 4 H), 1.99 (t, J = 11.4 Hz, 2 H), 2.05–2.29 (m, 4 H), 2.66 (tt, J = 6.8, 17.3 Hz, 4 H), 2.81 (dd, J = 5.1, 17.3 Hz, 1 H), 3.11 (t, J = 12.6 Hz, 1 H), 3.71 (t, J = 7.2 Hz, 2 H), 3.95–4.05 (m, 4 H), 4.38 (dt, J = 4.4, 12.7 Hz, 1 H). ^13^C NMR (126 MHz, CDCl_3_) δ 11.71, 12.66, 20.90, 21.33, 26.73, 27.85, 31.88, 35.75, 37.74, 41.97, 42.70, 46.97, 49.39, 52.17, 57.45, 64.09, 166.92, 193.55. LR–ESI–MS: calculated for C_20_H_37_N_3_O_3_S_2_ [M + H]^+^: 395.4, found: 396.7. HPLC: 7.618 min.

### Cell line and culture conditions

The fresh medium was configured at the composition of stock liquid: FBS: antibiotic = 90:10:1(v/v/v), and the stock liquid was configured by Grace’s Insect Medium with 0.35 g/L sodium bicarbonate. The growth status of *Sf9* cell was observed by IPCM. When the degree of cell fusion exceeded 80%, the old culture solution was discarded and the cells were rinsed with PBS buffer solution. After rinsing the cells, the PBS buffer solution was discarded, and fresh medium was added to pipette and resuspend the *Sf9* cell. Finally, the cells were removed from the ultra-clean bench and returned to a conventional biochemical incubator for incubation at 27 °C. Passaging the cells every 3 days to ensure that the cells are in the logarithmic growth phase as needed in the experiments.

### Evaluation of cells viability by 3-(4,5-dimethylthiazole-2yl)-2,5-diphenyl (MTT) assays

*Sf9* cell in good condition were selected to prepare a cell suspension with a density of 6 × 10^4^–8 × 10^4^ cells/mL. And then the *Sf9* cell were incubated in a 96-well plate with 100 µL cell suspensions in each well at 27 °C for 24 h. Different concentrations of test compound were added to the test group, and 1.0% DMSO was added to the control group. The experiment was set up with 6 repetitive experiments, and the data of 24, 48, 72 h were recorded. Adding 10 μL of MTT solution (prepared in PBS) to the corresponding wells, followed by incubation in the dark for 4 h in an incubator at a constant temperature. All liquid was discarded and 150 μL of DMSO was added to dissolve the crystalline formazan. After the crystals were completely dissolved, the 96-well plate was removed from the bench. The absorbance (OD value) of the corresponding well was detected at 429 mm with a microplate reader. Cell viability was calculated by the following equation: Inhibition rate (%) = (1 − OD_trest_)/(OD_control_) × 100%.

### Morphological observation by inverted phase contrast microscopy (IPCM)

1.5 ~ 2.0 mL of cell suspension was added to each well in a 6-well plate, and the cells entered the logarithmic growth phase after 24 h of culture. One well was set as the blank control group, and the test compound was added to the remaining wells. The 6-well plate was placed in a biochemical incubator to continue culturing the cells, and the cell morphology was observed and recorded with IPCM after 24, 48, and 72 h, respectively.

### Statistical analysis

The chemical structures were drawn by Chemdraw. Infrared spectras were processed by Origin 2019. Mass spectrums were processed by Qualitative Analysis of Mass Huter Acquisition Date. The NMR spectroscopys were analyzed by MestReNova. Single crystal diffraction data were obtained by Olex 2. The inhibition rates were performed using origin 9.0 and SPSS statistics software, and the standard deviation (SD) of six independent experiments were calculated. Histograms were drawn from the inhibition rates of *Sf9* cell induced by different concentrations of compounds at 24, 48, and 72 h. And a linear fit was performed on the inhibition rates at 24 h, 48 h, and 72 h induction time to determine the IC_50_ of the compounds on the *Sf9* cell.

## Supplementary information

Supplementary Figures.
