# Supplementary material for: Synthesis, characterization of two matrine derivatives and their cytotoxic effect on Sf9 cell of Spodoptera frugiperda
Source: Sci Rep. 2020 Oct 22;10:17999. doi: 10.1038/s41598-020-75053-1 (PMC7581774; doi:10.1038/s41598-020-75053-1)
Supplement: Supplementary file 1 — Supplementary Figures. [file 41598_2020_75053_MOESM1_ESM.pdf]

SUPPLEMENTARY INFORMATION

Synthesis, Characterization of Two Matrine Derivatives and Their Cytotoxic Effect on *Sf9* Cell of *Spodoptera frugiperda*

Huiqing He[1,+], Xiangjing Qin[2,+], Fangyun Dong[1], Jingmin Ye[1], Chunbao (Charles) Xu[3], Hanhui Zhang[1], Zhanmei Liu[1], Xiaojing Lv[1], Yuehua Wu[1], Xuhong Jiang[1,*], Xingan Cheng[1,4,*]

[a] *Institute of Natural Product Chemistry, College of Chemistry and Chemical Engineering, Zhongkai University of Agriculture and Engineering, Guangzhou, Guangdong, 510225, China*

[b] *CAS Key Laboratory of Tropical Marine Bio-resources and Ecology, Guangdong Key Laboratory of Marine Materia Medica, South China Sea Institute of Oceanology, Chinese Academy of Sciences (CAS), Guangzhou, 510301, China*

[c] *Department of Chemical and Biochemical Engineering, Western University, London, Ontario N6A5B9, Canada*

[d] *Institute of Plant Health, Zhongkai University of Agriculture and Engineering, Guangzhou, Guangdong, 510225, China*

[+] These authors contributed equally to this work.

*Corresponding authors. E-mail address:jiangxh69@163.com (X.H.Jiang) ;anzai_28@163.com(X.A.Cheng)

Content

Figure S1 LR-ESI-MS of Compound **1**

Figure S2 LR-ESI-MS of Compound **2**

Figure S3 ^{1}H NMR spectrum of Compound **1**

Figure S4 ^{13}C NMR spectrum of Compound **1**

Figure S5 ^{1}H NMR spectrum of Compound **2**

Figure S6 ^{13}C NMR spectrum of Compound **2**

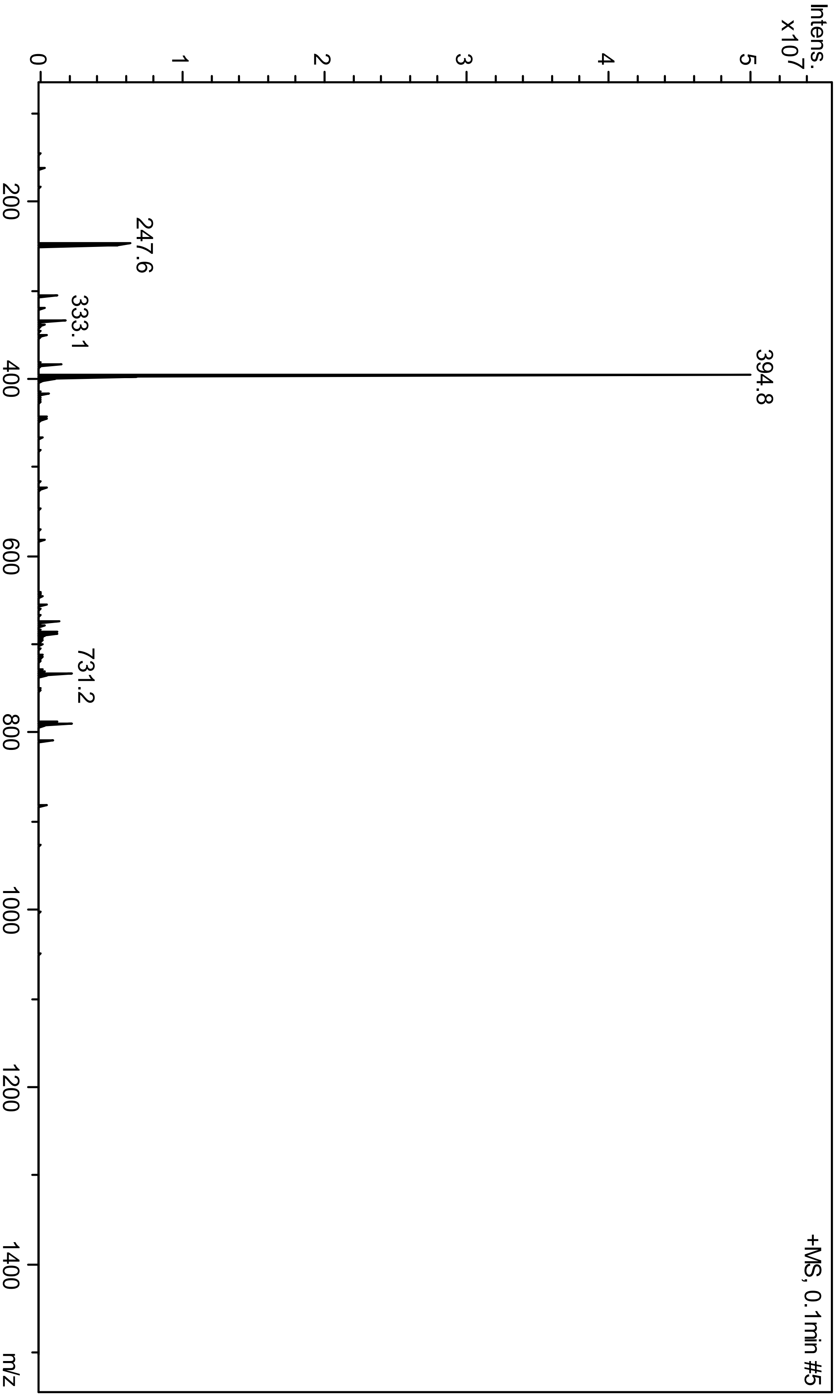

Figure S1 *LR-ESI-MS of Compound* ***1***

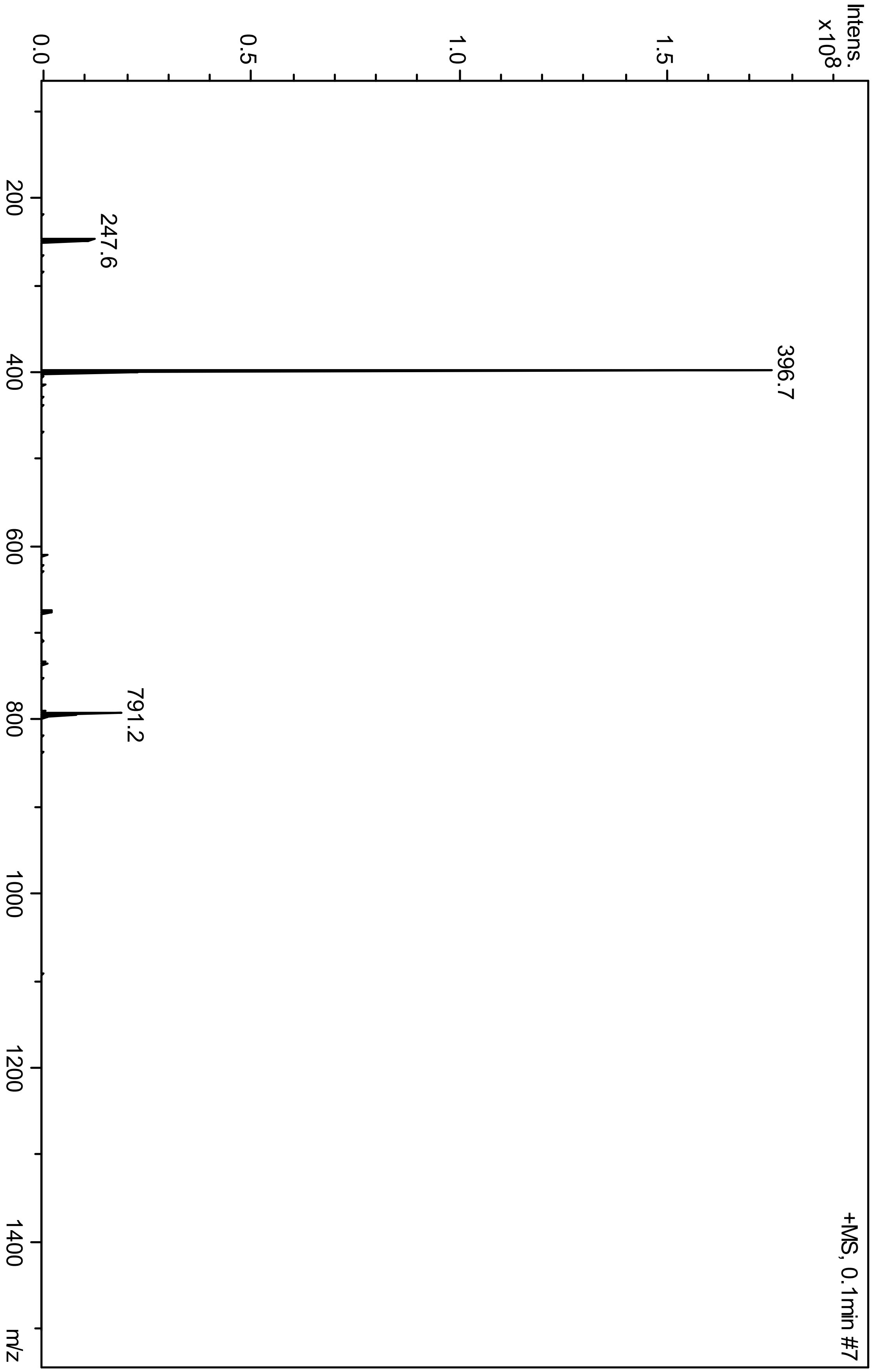

Figure S2 *LR-ESI-MS of Compound* ***2***

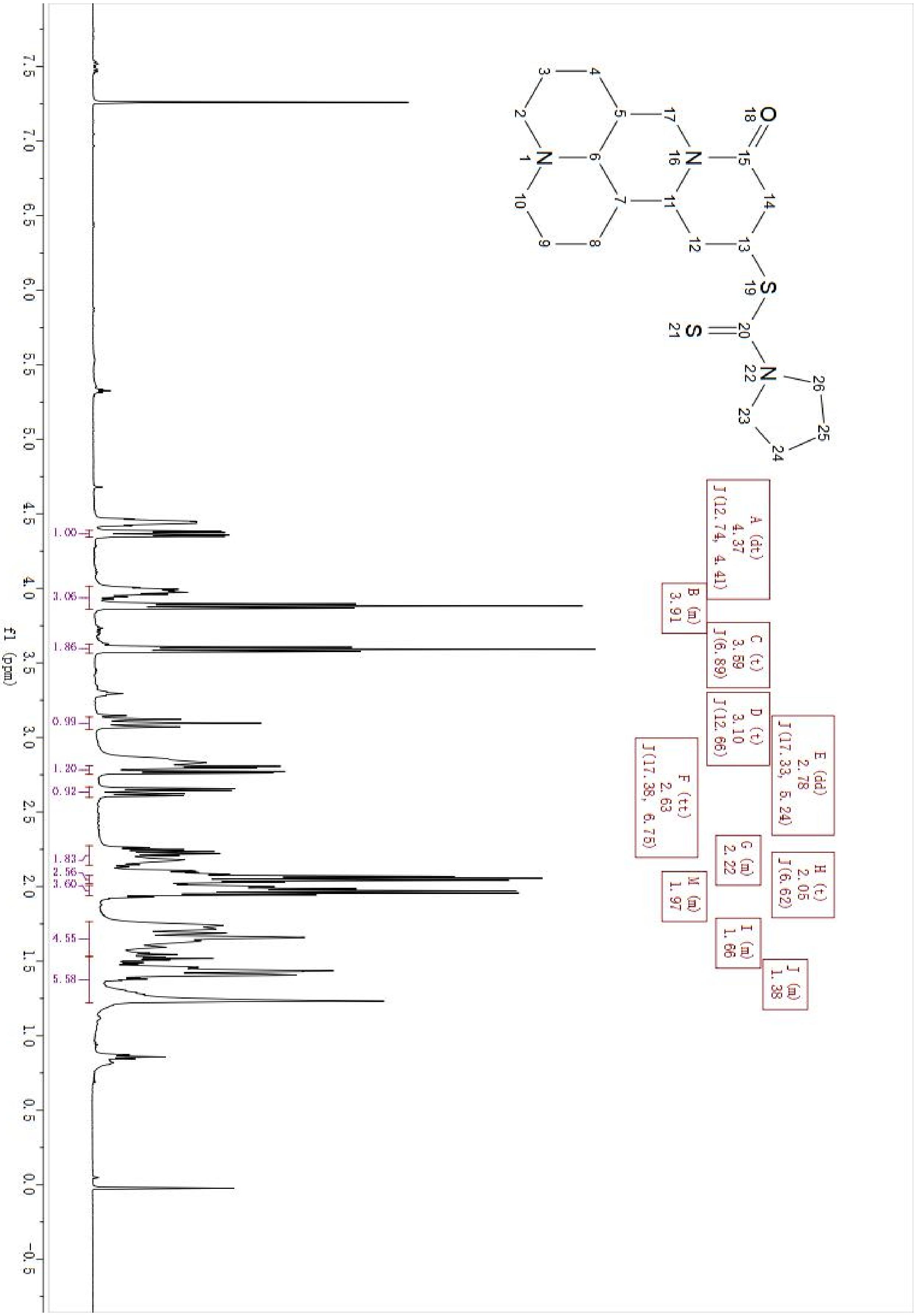

Figure S3 *¹H NMR spectrum of Compound* ***1***

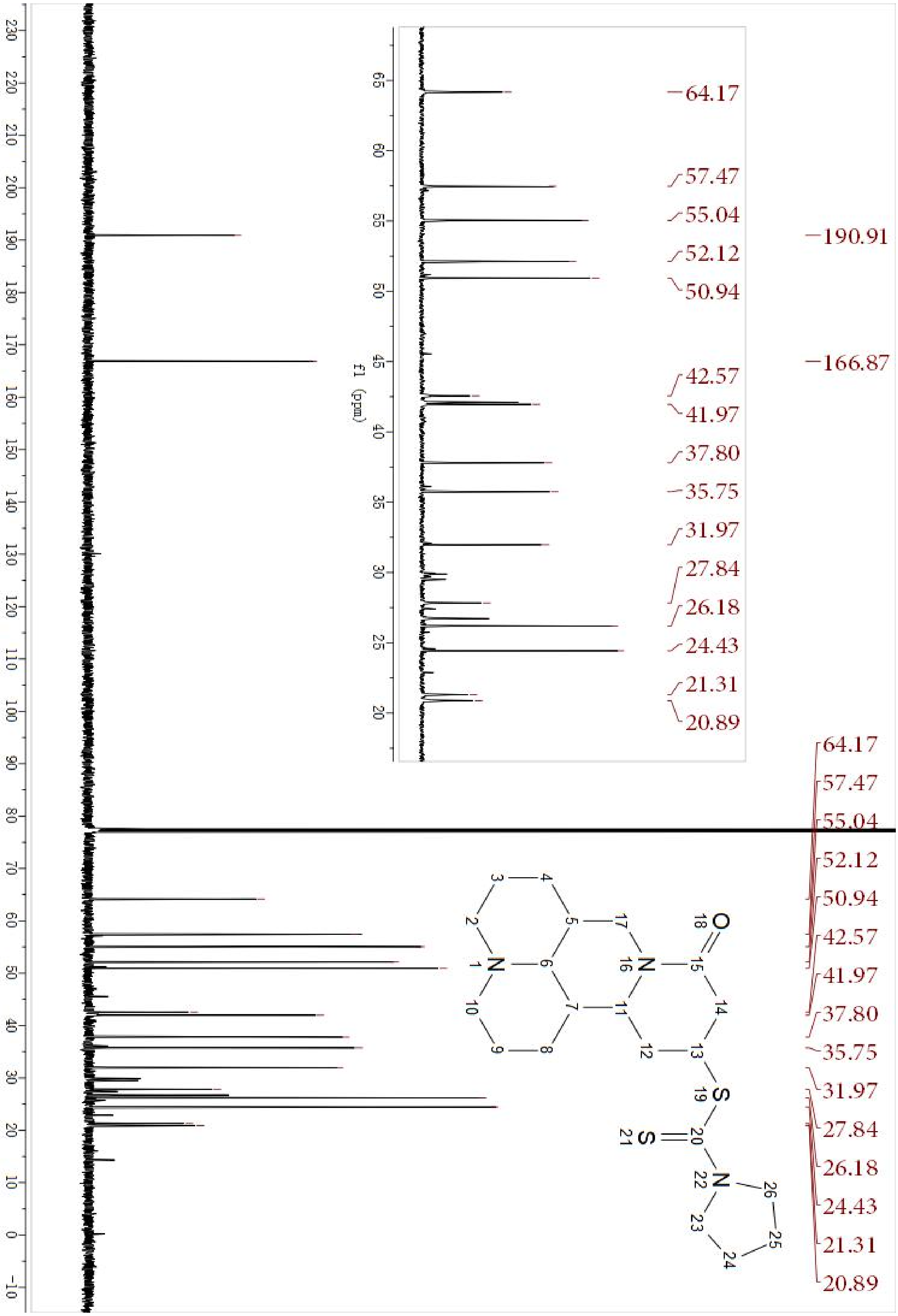

Figure S4 *^{13}C NMR spectrum of Compound* ***1***

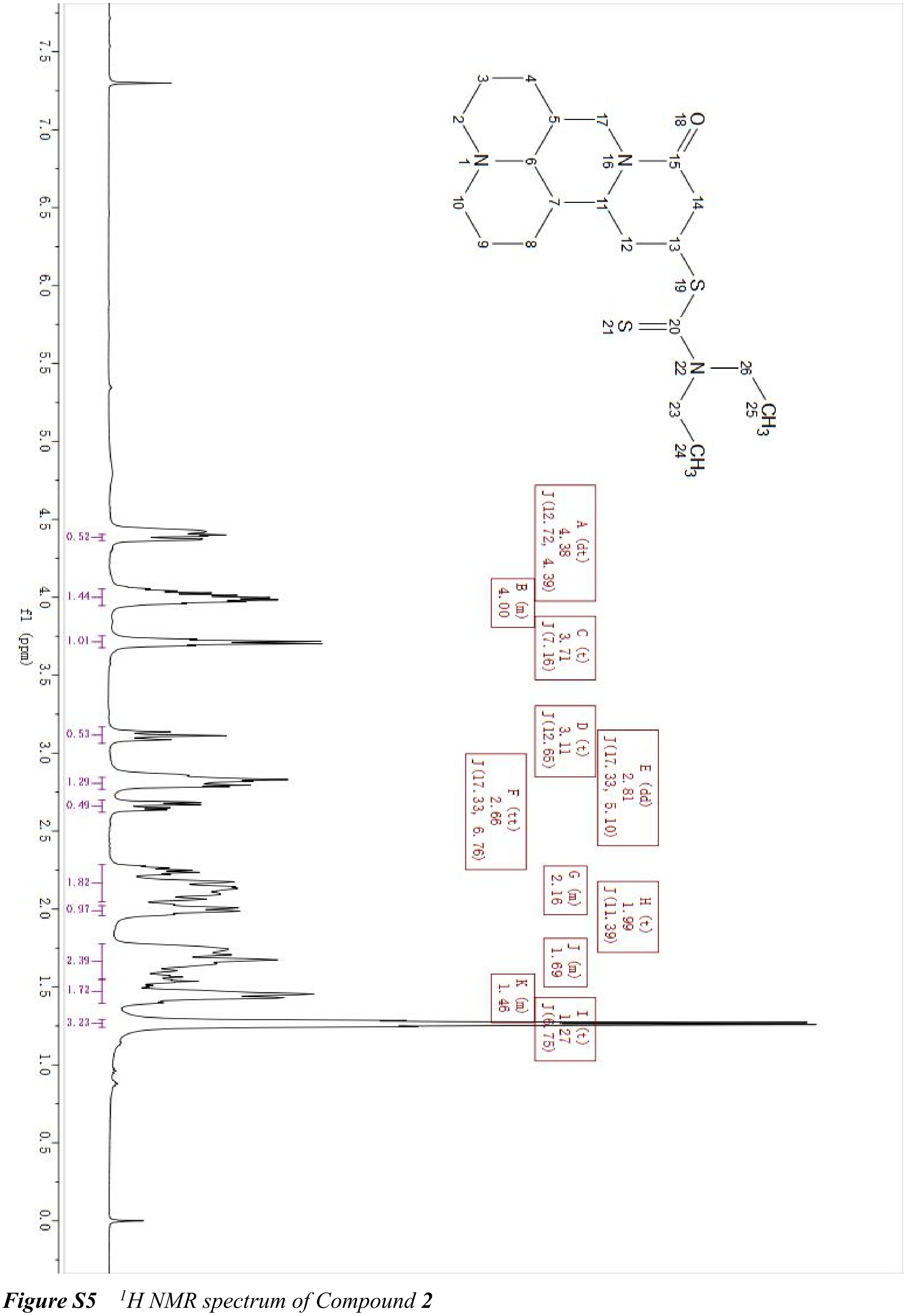

Figure S5 *^{1}H NMR spectrum of Compound* ***2***

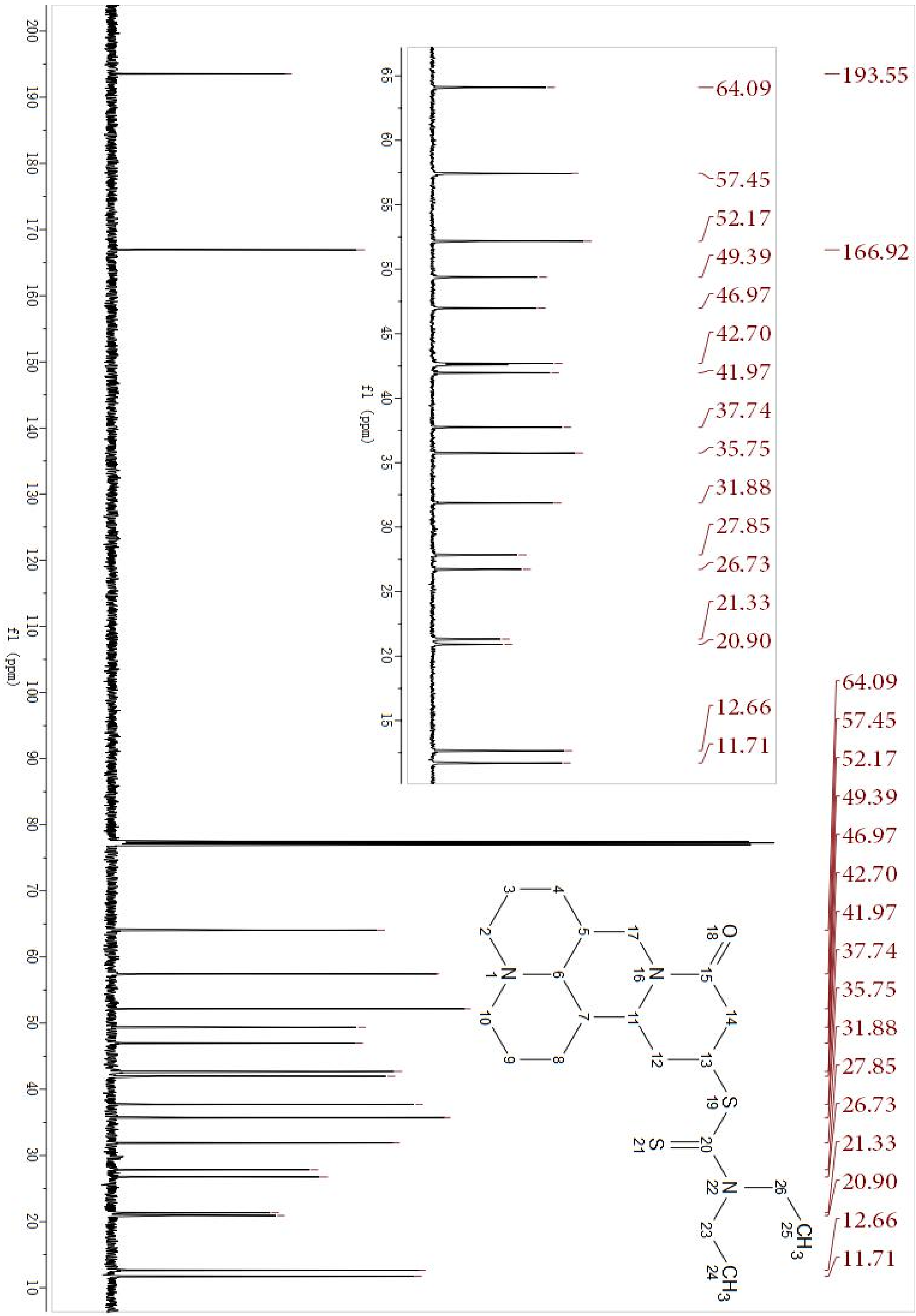

Figure S6 *^{13}C NMR spectrum of Compound* ***2***
